# A Nomogram Based on Apelin-12 for the Prediction of Major Adverse Cardiovascular Events after Percutaneous Coronary Intervention among Patients with ST-Segment Elevation Myocardial Infarction

**DOI:** 10.1155/2020/9416803

**Published:** 2020-02-06

**Authors:** Enfa Zhao, Hang Xie, Yushun Zhang

**Affiliations:** Department of Structural Heart Disease, The First Affiliated Hospital of Xi'an Jiaotong University, Xi'an 710061, China

## Abstract

**Objective:**

This study aimed to establish a clinical prognostic nomogram for predicting major adverse cardiovascular events (MACEs) after primary percutaneous coronary intervention (PCI) among patients with ST-segment elevation myocardial infarction (STEMI).

**Methods:**

Information on 464 patients with STEMI who performed PCI procedures was included. After removing patients with incomplete clinical information, a total of 460 patients followed for 2.5 years were randomly divided into evaluation (*n* = 324) and validation (*n* = 324) and validation (

**Results:**

Apelin-12 change rate, apelin-12 level, age, pathological Q wave, myocardial infarction history, anterior wall myocardial infarction, Killip's classification > I, uric acid, total cholesterol, cTnI, and the left atrial diameter were independently associated with MACEs (all *P* < 0.05). After incorporating these 11 factors, the nomogram achieved good concordance indexes of 0.758 (95%CI = 0.707–0.809) and 0.763 (95%CI = 0.689–0.837) in predicting MACEs in the evaluation and validation cohorts, respectively, and had well-fitted calibration curves. The decision curve analysis (DCA) revealed that the nomogram was clinically useful.

**Conclusions:**

We established and validated a novel nomogram that can provide individual prediction of MACEs for patients with STEMI after PCI procedures in a Chinese population. This practical prognostic nomogram may help clinicians in decision making and enable a more accurate risk assessment.

## 1. Introduction

Cardiovascular disease (CVD) is the main cause of death and disease burden in China and throughout the world, despite the technological advancement and the increasing level of awareness [1, 2]. Percutaneous coronary intervention (PCI) provides normal blood flow in the responsible artery in patients with STEMI, contributing remarkably to the regression of symptoms and better prognosis [3]. The preferred reperfusion therapy for STEMI is primary PCI [4]. It was reported that STEMI following successful PCI is the primary cause of mortality and morbidity worldwide in MACEs as a consequence of acute heart failure, mechanical complications, and cardiac shock after procedure [5]. It was revealed that many clinical, biochemical, and echocardiographic factors influenced the prognosis of STEMI following PCI [6]. Several tools for cardiovascular disease risk and prognosis evaluation in different populations have been established to guide clinical practice. The widely used tools included the Framingham general CVD equations in the United States, the QRISK in the United Kingdom, the Systematic Coronary Risk Evaluation model in Europe, and the Pooled Cohort Equations for atherosclerotic CVD (ASCVD) released in the American Heart Association guideline, as well as Thrombolysis in Myocardial Infarction (TIMI) risk score, TIMI risk index, Evaluation of Methods and Management of Acute Coronary Events (EMMACE), Global Registry of Acute Coronary Events (GRACE), Primary Angioplasty in Myocardial Infarction (PAMI), and Canada Acute Coronary Syndrome (C-ACS) Risk Score [7–16]. However, these equations presented above were all derived from Western populations, which limited their application to other populations. It is known that various lifestyle factors, an aging population, and longer life spans among different populations have led to distinct CVD outcome during the past decade [8, 17, 18].

In recent years, numerous novel markers of cardiovascular disease have been used in clinical practice. Novel and reliable biomarkers are urgently needed to incorporate within clinical model to help clinicians to both identify patients at high risk for adverse clinical prognosis and provide them with proper prevention program by more accurate prognosis estimation. Apelin, a 77-amino acid peptide secreted by white adipose tissue, functions as the endogenous ligand for the human orphan G protein-coupled receptor (APJ) [19]. It was revealed that the apelin-APJ system plays an essential role in the cardiovascular system, heart development, and was related inversely to the process of arterial atherosclerosis [20–23]. Apelin-12, one of the most potent apelin peptides, is involved in the regulation of body fluid homeostasis and has a positive inotropic action in failing myocardium [24, 25]. A clinical study indicated that the plasma apelin-12 concentration is decreased early after acute myocardial infarction (AMI) and remains remarkably below baseline at 24 weeks [26]. It was suggested that the apelin-APJ system may be a promising tool in the diagnosis and treatment of CVD. However, the present models seldom incorporated the apelin-12 into prediction equations. Therefore, the objective of this study was to develop and validate a clinical nomogram after incorporating the apelin-12 for prediction of MACEs in patients with STEMI after primary PCI using publicly data repository.

## 2. Materials and Methods

### 2.1. Data Sources and Study Population

The Dryad Digital Repository (https://datadryad.org/) is a curated resource that allows the data underlying scientific publications to be discoverable, citable, and freely reusable to create knowledge. Clinical information of 464 patients with STEMI who performed PCI procedures was downloaded from Dryad data repository (https://doi.org/10.5061/dryad.pf56m). The original study was published previously to explore factors predicting the probability of MACEs after primary PCI in patients with STEMI [6]. After screening the original data, a total of 32 variables were selected for further analysis. Four patients with incomplete clinical information were excluded from this study, and 460 remained. Patients followed up for 2.5 years were randomly divided into evaluation (*n* = 324) and validation (*n* = 136) cohorts based on a computer-generated allocation sequence. Since the application of data complied with the Dryad publication guidelines, the approval of institutional ethics committees was not required in this study.

### 2.2. Clinical Outcomes Definitions

A MACE is defined as the end point of this study, which referred to the composite of cardiac death, clinically driven target lesion revascularisation, recurrent target vessel myocardial infarction, cardiogenic shock, or demonstrated congestive heart failure. The apelin change rate was defined as the level of apelin-12 at 72 hours after PCI compared with that immediately before PCI. The other clinical outcomes were defined in a previous study [6]. All patients had a clinical follow-up for a 30-month period after operation.

### 2.3. Identification of Candidate Clinicopathological Variables and Nomogram Development

A total of 32 potential variables were included in this study. The associations of these variables with MACEs were identified using Cox proportional hazards regression models. Backward stepwise selection with the Akaike information criterion (AIC) was used to select variables for the multivariable Cox proportional hazards regression models [27]. Results are reported as hazard ratios (HRs) and 95% CIs. Clinicopathological variables with the *P*-value of ≤0.05 were included in the model. The identified variables based on the results of multivariate analysis were incorporated in the nomogram to predict the risk of 1-year and 2-year MACEs after PCI using statistical software (rms in R, version 3.5.1; http://www.r-project.org). The fitted nomogram used the covariates as input and generated a risk score for each patient.

### 2.4. Assessment and Validation of the Nomogram

The discrimination and calibration power are two important aspects of the performance of the established nomograms, and they were evaluated by using the concordance index (C-index) and calibration curves, both in evaluation and validation cohorts, respectively. The calibration of the nomogram was assessed by comparing the nomogram-predicted MACEs probability with the observed Kaplan-Meier estimates of MACEs probability. In a perfectly calibrated curve, the predictions should fall on the diagonal 45° line of the calibration plot. Calibration estimates how close the nomogram estimated probability is to the observed probability. A risk score for each patient was generated from the nomogram, which was calculated as a linear combination of the selected variables that were weighted by their respective regression coefficients of the multivariate Cox proportional hazards regression analysis conducted in the evaluation cohort to reflect the probability of MACEs. All patients were divided into two groups (a high-risk group and a low-risk group) according to the median risk score. Patients were further stratified into two subgroups according to the median value of apelin-12 level on admission. The Kaplan-Meier method was used to compare the difference in prognosis between the high- and low-risk groups using the evaluation and validation cohorts.

### 2.5. Clinical Usefulness of the Nomogram

The clinical usefulness of the nomogram was estimated using the decision curve analysis (DCA) by quantifying the net benefits for a range of threshold probabilities using the combined evaluation and validation cohorts [28]. The net benefit was calculated by subtracting the ratio of all patients who are false positive from the fraction of the individuals who are true positive and by weighing the relative harm of forgoing interventions compared with the negative results of an unnecessary intervention.

## 3. Results

### 3.1. Clinicopathologic Characteristics

The basic characteristics of the patients in the evaluation and validation cohorts are summarized in Table 1. There are no significant differences between the two cohorts in MACEs prevalence (*P*=0.3634). MACEs were presented in 26.85% and 22.79% in the evaluation and validation cohorts, respectively. The baseline clinicopathologic characteristics were similar between the two cohorts, except for Killip's classification > I (*P*=0.0018).

### 3.2. Nomogram Building

**Figure 1:**
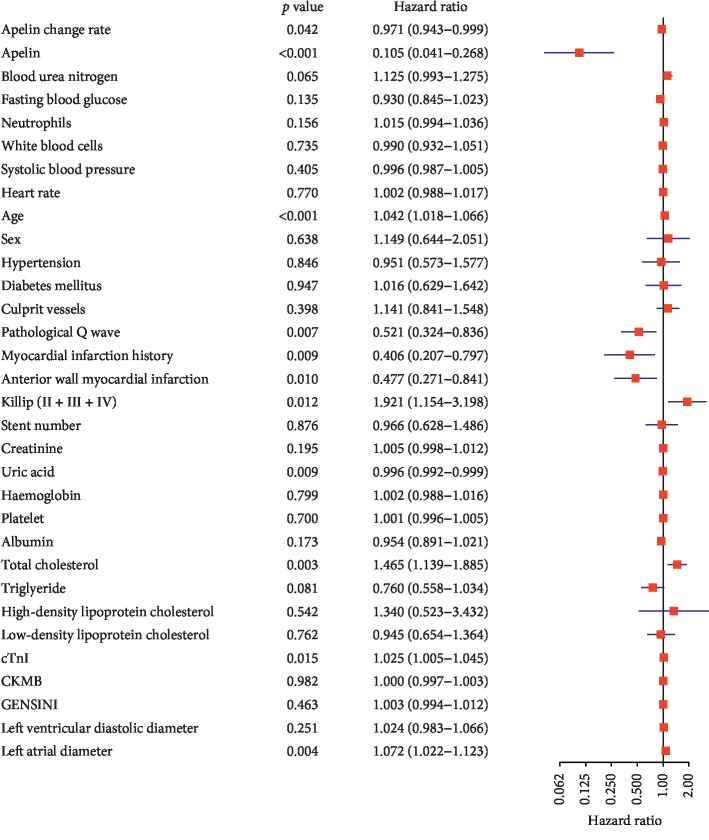
Multivariate Cox proportional hazards regression analysis showing the association of variables with major adverse cardiovascular events.

**Figure 2:**
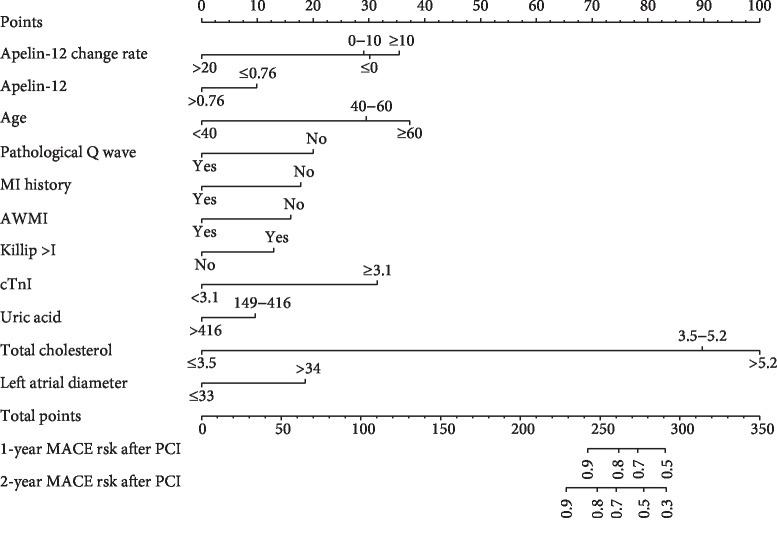
Nomogram predicting 1- and 2-year major adverse cardiovascular events probability for patients with ST-segment elevation myocardial infarction after primary percutaneous coronary intervention. The nomogram allows the clinician to determine the probability of the 1-year and 2-year risk for an individual patient using a combination of covariates. Using the patient's age, you can draw a vertical line from that variable to the points scale. After repeating the process for each variable, the scores for each variable can be summed and located on the “Total Points” axis. Finally, a vertical line can be drawn straight down from the plotted total point axis to the probability axis to locate the likelihood of 1-year and 2-year risk.

According to the multivariate Cox proportional hazards regression analysis, 11 candidate clinical variables were found to meet the threshold of *P* < 0.05. The multivariate logistic regression analysis showed that apelin-12 change rate (HR = 0.970, 95%CI = 0.943–0.999, *P*=0.042), apelin-12 level (HR = 0.105, 95%CI = 0.041–0.268, *P* < 0.001), age (HR = 1.042, 95%CI = 1.018–1.066, *P* < 0.001), pathological Q wave (HR = 0.521, 95%CI = 0.324–0.836, *P*=0.007), myocardial infarction history (HR = 0.406, 95%CI = 0.207–0.797, *P*=0.009), anterior wall myocardial infarction (HR = 0.477, 95%CI = 0.271–0.841, *P*=0.010), Killip's classification > I (HR = 1.921, 95%CI = 1.154–3.198, *P*=0.012), uric acid (HR = 0.996, 95%CI = 0.992–0.999, *P*=0.009), total cholesterol (HR = 1.465, 95%CI = 1.139–1.885, *P*=0.003), cTnI (HR = 1.025, 95%CI = 1.005–1.045, *P*=0.015), and the left atrial diameter (HR = 1.072, 95%CI = 1.022–1.123, *P*=0.004) were independently associated with MACEs (Figure 1). The predictive nomogram that integrated all the significant independent predictors for the MACEs rate was then developed in the evaluation cohort (Figure 2). The formula for calculating the total point of the nomogram is as follows: 1.0304 ∗ (apelin change rate) + 9.5522 ∗(apelin) + 0.9600 ∗ (age) + 1.9201 ∗ I (pathological Q wave) +2.4603 ∗ I (myocardial infarction history) + 2.0949 ∗ I (anterior wall myocardial infarction) + 0.5206 ∗ I (Killip's classification > I) + 1.0043 ∗ (uric acid) + 0.6826 ∗ (total cholesterol) + 0.9756 ∗ (cTnI) + 0.9332 ∗ (left atrial diameter). The indicator function (I) equals 1 if the statement in the parentheses is true and is equal to 0 otherwise.

### 3.3. Assessment of the Nomogram Performance

**Figure 3:**
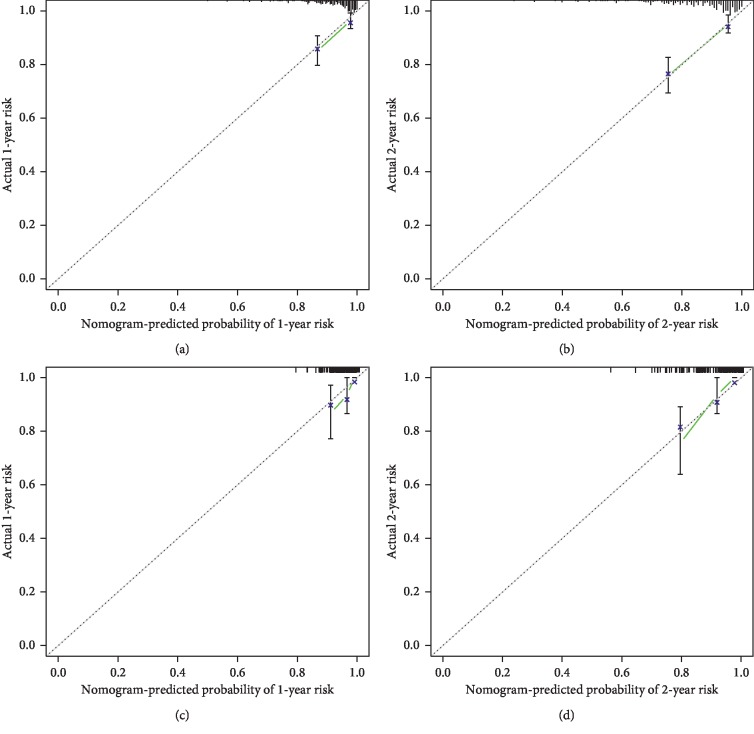
The calibration curve for predicting major adverse cardiovascular events probability at (a) 1 year and (b) 2 years in the evaluation cohort and at (c) 1 year and (d) 2 years in the validation cohort. Nomogram-predicted probability of major adverse cardiovascular events is plotted on the *X*-axis; actual probability is plotted on the *Y*-axis.

The nomogram yielded a C-index of 0.758 (95%CI = 0.707 to 0.809) using the evaluation cohort. A widely accepted approach demonstrates that a C-index of more than 0.75 reveals clearly useful discrimination [29, 30]. Therefore, the established nomogram presented satisfactory discrimination in the evaluation cohort. The calibration curves for the MACEs probability at 1 and 2 years after PCI showed favorable agreement between the nomogram-based prediction and actual observation, demonstrating good calibration of the nomogram (Figures 3(a)–3(b)).

### 3.4. Validation of the Nomogram in the Validation Cohort

The fitted nomogram used the covariates as input and generated a risk score for each patient in both cohorts. The formula for calculating the risk score is as follows: 0.4215 ∗ (apelin) − 0.3589 ∗ (apelin change rate) + 0.4587 ∗ (age) − 0.7173 ∗ I (pathological Q wave) − 0.6211 ∗ I (myocardial infarction history) − 0.6143 ∗ I (anterior wall myocardial infarction) + 0.5037 ∗ I (Killip's classification > I) − 0.3868 ∗ (uric acid) + 0.4065 ∗ (total cholesterol) + 1.2534 ∗ (cTnI) + 0.7149 ∗(left atrial diameter). The indicator function (I) equals 1 if the statement in the parentheses is true and is equal to 0 otherwise. The favorable calibration of the nomogram was confirmed in the validation cohort (Figures 3(c)–3(d)). Furthermore, the nomogram also exhibited good discrimination with a C-index of 0.763 (95% CI, 0.689–0.837) in the validation cohort. As a result, the nomogram performed well using both the evaluation and validation cohorts. We further compare the performance of the developed risk score if apelin levels (apelin and Δapelin) are not incorporated in the calculation. The nomogram without including apelin levels (apelin and apelin change rate) yielded a C-index of 0.722 (95% CI = 0.671 to 0.772), which was inferior to that obtained from the C-index of 0.758 (95% CI = 0.707 to 0.809) in the evaluation cohort and the C-index of 0.763 (95% CI, 0.689–0.837) in the validation cohort in the present nomogram. This revealed that apelin levels improve the predictive power of the risk score.

### 3.5. Survival Analysis between High- and Low-Risk Groups

**Figure 4:**
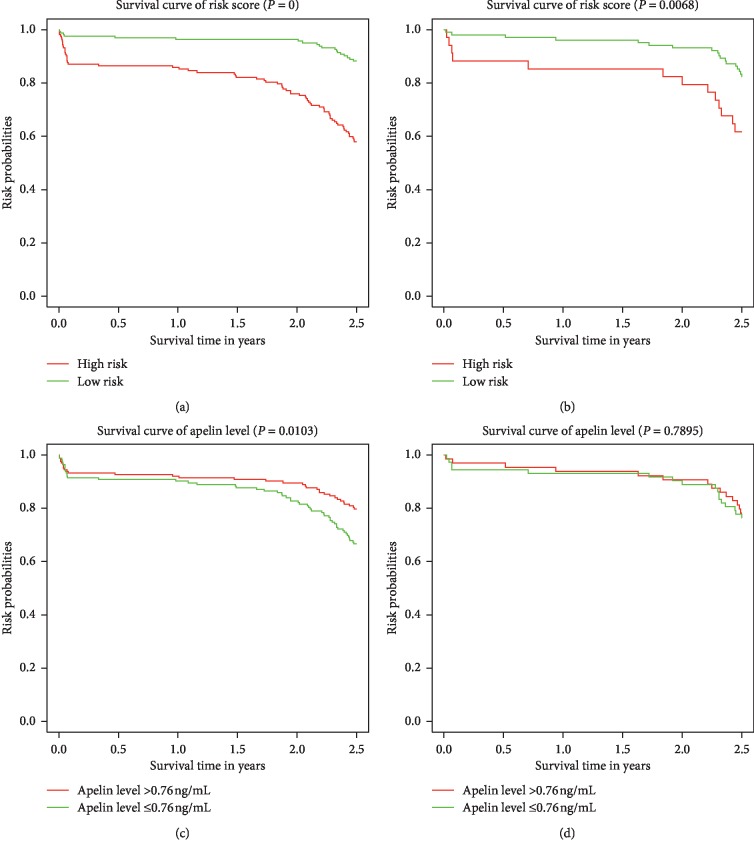
Kaplan-Meier survival curves of the evaluation and validation cohorts categorized into low- and high-risk groups. A significant association between the risk score and MACEs was observed in the evaluation cohort (a) and confirmed in the validation cohort (b). Survival curves between the higher apelin-12 group (>0.76 ng/mL) and lower apelin-12 group (≤0.76 ng/mL) in the evaluation cohort (c) and the validation cohort (d).

After gaining the risk scores from the nomogram, the patients were classified into a low-risk group or a high-risk group using the median risk score as the cutoff value. Kaplan-Meier curves and log-rank test analysis in patients of the low-risk group and high-risk group during 2.5-year follow-up are shown in Figure 4. As revealed in Figure 4(a), clear discrimination between the MACEs of the high- and low-risk patients was observed in the evaluation cohort, which was confirmed in the validation cohort (Figure 4(b)). Based on the median value of apelin-12 on admission, patients were classified into a higher group (>0.76 ng/mL) and a lower group (≤0.76 ng/mL). Significant differences in survival curves were noted between patients with different apelin-12 (*P*=0.0103, Figure 4(c)). However, such differences were not observed in the validation cohort (Figure 4(d)), which may be due to the relatively small sample size. Therefore, our nomogram can successfully distinguish patients with high risk of MACEs after PCI from those with low risk.

### 3.6. Clinical Usefulness of the Nomogram and Comparing the Performance of the Newly Developed Risk Score with the Already Available Risk Scores

**Figure 5:**
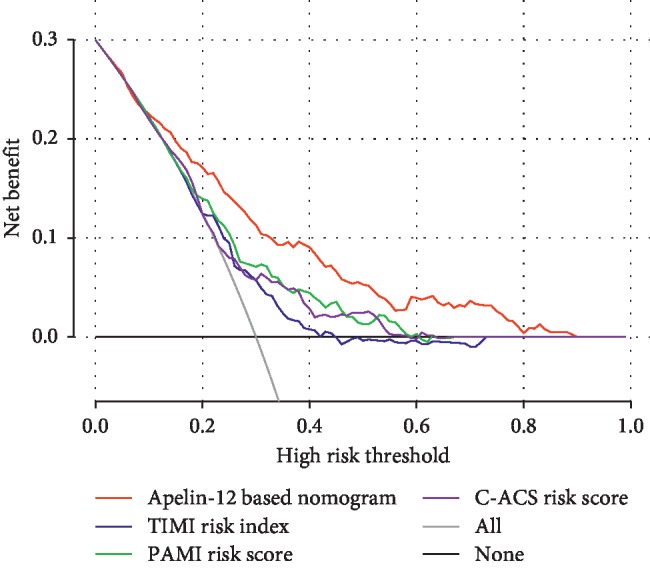
Decision curve analysis of apelin-12 based nomogram and other already available risk scores in terms of major adverse cardiovascular events risk in the whole cohort. The *X*-axis represents the threshold probability. The *Y*-axis measures the net benefit. The red line illustrates the major adverse cardiovascular events risk nomogram. The threshold probability is where the expected benefit of treatment balances the expected benefit of avoiding treatment.

The decision curve analysis is a novel method that evaluates predictive models from the perspective of clinical consequences. The threshold probability is where the expected benefit of treatment balances the expected benefit of avoiding treatment. When the threshold probability ranged from 0.01 to 0.86 in the combined evaluation and validation cohorts, using the apelin-12 based nomogram to predict MACEs yields a greater net benefit than the treat-all or treat-none strategies. For example, if the possibility of MACEs in a patient is over the threshold probability, then a treatment strategy should be adopted. Therefore, the decision curve analysis indicated that the nomogram is clinically useful. Moreover, the newly developed nomogram in this study also displayed more powerful efficiency of the discrimination for MACEs prediction in the whole cohort compared with the other available risk scores systems (Figure 5).

In addition, we compared the discrimination of the nomogram with that of other already available risk scores to predict MACEs in the evaluation and validation data sets. The apelin-12 based nomogram discrimination for MACEs prediction was superior to that of the other already available risk scores in the evaluation cohort. The discrimination of the nomogram for MACEs prediction was also enhanced compared with the available risk scores in the validation cohort (Table 2).

## 4. Discussion

Apelin is the endogenous ligand of APJ, which is commonly expressed in several organs and tissues, such as the heart, kidney, lung, and adipose tissue [31]. Apelin and APJ receptor play a vital role in the cardiovascular development and may also be involved in the cardiovascular pathological processes [32–34]. A recent study found that apelin had biological functions such as peripheral and coronary vasodilation [35]. It was shown that intravenous apelin administration in rodents decreases systemic venous tone and mean arterial pressure [36, 37]. In patients with stable angina, plasma apelin was found to be negatively correlated with the coronary artery stenosis severity independent of other cardiovascular risk factors [38]. The clinical prognosis in patients with STEMI after PCI is closely associated with the apelin-12 concentration on admission [6]. All of the above studies indicated that apelin may play an important role in cardiovascular diseases. However, as far as we know, at present there is no clinical prediction model which incorporated apelin-12 to predict MACEs in patients with STEMI after PCI. Therefore, a well-performed risk prediction tool incorporating apelin-12 is greatly needed.

It was known that nomograms are commonly used as a prognostic tool in oncology and medicine. They provided individual predictions of future clinical outcomes by combining the effects of various variables associated with these events. As far as we know, this is the first clinical prediction model incorporating apelin-12 for predicting MACEs in patients with STEMI after PCI in a Chinese population. In this study, we have constructed and validated a relatively accurate clinical nomogram, which demonstrated adequate discrimination and calibration power to provide an individualized estimation for the MACEs risk at 1 and 2 years in STEMI patients after PCI. For the construction of the nomogram, 11 significant predictors were screened by the multivariate Cox proportional hazards regression analysis. They were also used to construct the apelin-related nomogram and has presented favorable discrimination and diagnostic value to predict the MACEs risk of patients with STEMI after PCI in the evaluation cohort (C-index: 0.758, 95%CI: 0.707–0.809). The validation cohort further confirmed the clinical significance of the nomogram with the C-index of 0.763 (95CI%: 0.689–0.837), which demonstrated an advantage of individual prediction of the MACEs risk in STEMI patients after PCI. We further compared the discrimination of the nomogram with that of the other already available risk scores to predict MACEs in the two cohorts. The apelin-12 based nomogram discrimination for MACEs prediction was superior to that of the other already available risk scores in both cohorts. These results indicate that these risk scores being derived from Western populations may limit their application in Chinese populations. However, it is still not easy to choose when to use the nomogram. The role of the clinical decision curve analysis is to select the optimal schedule of treatment via analyzing all potential behaviors and outcomes in the clinical decision making process. In the study, according to the results of the DCA related to the apelin-based nomogram, when the threshold probability is >1% and <86%, the use of the nomogram would provide more benefit than either the treat-all-patients approach or the treat-none approach. Furthermore, the newly developed nomogram also displayed more powerful efficiency of discrimination for MACEs prediction in the whole cohort compared with the other available risk scores systems.

When it comes to the clinical application of the nomogram, we can take a 65-year-old male patient who has recently been diagnosed with STEMI with Killip's classification of III and then performed PCI procedure. The patient presented a history of pathological Q wave, anterior wall myocardial infarction, and left atrial diameter of 35 mm. His apelin-12 level immediately before PCI was 0.79 ng/mL and 72 hours after PCI was 12.36 ng/mL. The lab examination parameters of uric acid, triglyceride, and peak cTnI were 452 mmol/L, 7.66 mmol/L, and 38.5 ng/mL, respectively. He wonders about the probability of MACEs of 1-year and 2-year risk. Using the patient's age, you can draw a vertical line from that variable to the points scale. After repeating the process for each variable, the scores for each variable can be summed and located on the “Total Points” axis. Finally, a vertical line can be drawn straight down from the plotted total point axis to the survival axis to locate the likelihood of 1-year and 2-year probability. Furthermore, the nomogram successfully stratified STEMI patients into high- and low-risk groups, and the high-risk group revealed a significantly lower probability of MACEs. Therefore, our nomogram may act as a precise and reliable predictive model for MACEs in patients with STEMI after PCI in Chinese populations, which may contribute to patient management.

To improve the primary prevention and management of cardiovascular diseases, several tools have been developed to predict the probability of cardiovascular disease risk in different populations [8, 9, 18, 39]. However, they were mainly related to prevention of atherosclerotic cardiovascular disease, prediction of total fatal cardiovascular risk, and development of a cardiovascular disease risk algorithm. Besides, the variables varied in different models, and apelin-12 especially has never been used in a clinical nomogram. Therefore, we developed such nomogram for predicting the MACEs of STEMI patients after PCI. However, the limitations of the study should be considered. Firstly, the clinical variables used as potential predictors for MACEs were based on the Dryad data repository. It was difficult to acquire detailed information of all patients. Therefore, we failed to include other potential risk factors in our study, such as left ventricular ejection fraction. Besides, although our apelin-based prediction model confirmed favorable predictive power for MACEs, a multicenter validation study should be conducted to confirm the performance of the clinical nomogram in future investigations.

## 5. Conclusions

In summary, we developed and validated a nomogram incorporating both apelin-12 and clinical risk factors to predict MACES in patients with STEMI after PCI in a Chinese population. Our nomogram showed a satisfactory performance, with a C-index of 0.763. This nomogram can be a precisely individualized predictive tool for prognosis. However, additional studies are needed to determine whether it can be applied to other populations before its implementation into clinical practice.

## Figures and Tables

**Table 1:** Participant characteristics in evaluation and validation cohorts.

Variables	Evaluation cohort (*n* = 324)	Validation cohort (*n* = 136)	*P*-value
Age (years)	62.60 ± 12.00	63.73 ± 11.70	0.3537
Male, *n* (%)	250 (77.16%)	103 (75.74%)	0.7425
Killip's classification > I, *n* (%)	92 (28.40%)	20 (14.71%)	0.0018
Diabetes mellitus, *n* (%)	99 (30.56%)	49 (36.03%)	0.2523
Hypertension, *n* (%)	195 (60.19%)	69 (50.74%)	0.0617
Myocardial infarction history, *n* (%)	37 (11.42%)	18 (13.24%)	0.5834
Anterior wall myocardial infarction, *n* (%)	160 (49.38%)	69 (50.74%)	0.7903
Apelin-12 (ng/mL)	0.82 ± 0.33	0.84 ± 0.35	0.5605
Apelin-12 change rate (%)	13.74 ± 8.98	12.94 ± 8.63	0.3783
SBP(mmHg)	131.34 ± 27.20	133.13 ± 27.10	0.5194
Albumin (g/L)	37.95 ± 3.92	37.87 ± 3.64	0.8385
Haemoglobin (g/L)	143.46 ± 17.39	144.65 ± 16.79	0.499
Total cholesterol (mmol/L)	5.67 ± 1.11	5.65 ± 1.06	0.8583
Triglyceride (mmol/L)	1.09 ± 0.85	1.16 ± 0.82	0.4159
High-density lipoprotein-C (mmol/L)	1.19 ± 0.27	1.24 ± 0.26	0.0676
Low-density lipoprotein-C (mmol/L)	3.06 ± 0.71	3.00 ± 0.76	0.4184
Fasting blood glucose (mmol/L)	7.63 ± 2.52	7.76 ± 2.57	0.6159
White blood cells × 10^9^/L	9.95 ± 3.72	10.40 ± 3.44	0.2269
Heart rate	77.54 ± 16.97	75.42 ± 17.56	0.2268
Neutrophil (%)	75.2 ± 11.71	76.89 ± 11.03	0.1515
Creatinine (mmol/L)	75.40 ± 25.20	72.91 ± 15.58	0.2855
Uric acid (mmol/L)	339.19 ± 75.20	332.5 ± 70.63	0.376
Platelet × 10^9^/L	232.59 ± 56.05	231.14 ± 57.02	0.8012
Blood urea nitrogen (mmol/L)	6.66 ± 2.05	6.89 ± 2.12	0.2776
Peak creatine kinase MB (U/L)	126.83 ± 91.26	130.85 ± 85.75	0.661
Peak cTnI (ng/ML)	16.66 ± 12.87	16.47 ± 12.86	0.8852
Pathological Q wave, *n* (%)	154 (47.53%)	67 (49.26%)	0.735
GENSINI score	72.92 ± 32.17	70.18 ± 32.18	0.405
Left atrial diameter (mm)	37.53 ± 5.59	37.17 ± 5.90	0.5356
Left ventricular diastolic diameter (mm)	50.36 ± 6.24	50.51 ± 6.36	0.8151

**Table 2:** Comparisons of C-indexes of the present risk score with other already available risk scores to predict major adverse cardiovascular events in the evaluation and validation data sets.

Risk scores	Evaluation cohort		Validation cohort	
	C-index	95%CI	C-index	95%CI
Apelin-12 based nomogram	0.758	0.707–0.809	0.763	0.689–0.837
TIMI risk index	0.625	0.568–0.682	0.587	0.489–0.685
PAMI risk score	0.652	0.593–0.711	0.657	0.559–0.755
C-ACS risk score	0.638	0.579–0.697	0.614	0.514–0.714

TIMI = thrombolysis in myocardial infarction; PAMI=primary angioplasty in myocardial infarction; C-ACS =Canada acute coronary syndrome; CI = confidence interval.

## Data Availability

The data used to support the findings of this study are available from the corresponding author upon request.
